# Effect of Tumor Stage and Molecular Subtypes on Five-Year Overall Survival in Breast Cancer: A Single-Center Study From Pakistan

**DOI:** 10.7759/cureus.110274

**Published:** 2026-06-04

**Authors:** Mohammad A Sahi, Abdul S Waqar, Anam Siddique, Jannat Asif, Ayesha Waqar, Shahid Rasul

**Affiliations:** 1 Oncology, Cancer Care Hospital and Research Centre, Lahore, PAK; 2 Medicine, Chaudhry Muhammad Akram Teaching and Research Hospital, Lahore, PAK

**Keywords:** breast cancer, cox regression, her2, kaplan–meier, molecular subtype, overall survival, pakistan, radiation & medical oncology

## Abstract

Background

Breast cancer is a biologically diverse malignancy in which stage at diagnosis and receptor-defined molecular subtype are important determinants of prognosis. In Pakistan, delayed presentation remains common, partly because of limited awareness, socioeconomic barriers, and variable access to diagnostic and treatment services. This study evaluated five-year overall survival (OS) by tumor stage and molecular subtype in a curative-intent breast cancer cohort from a tertiary cancer center in Pakistan.

Methodology

We retrospectively reviewed women diagnosed in 2020 with biopsy-confirmed invasive breast cancer, clinical Stage I-III disease, no distant metastasis (M0), and treatment with curative intent. OS was measured from the date of diagnostic biopsy to death from any cause or last documented follow-up. Survival was estimated using the Kaplan-Meier method, group differences were assessed with log-rank testing, and independent prognostic factors were evaluated using multivariable Cox regression, including stage, molecular subtype, age, and tumor grade. Treatment characteristics, including surgery type, chemotherapy timing, and radiotherapy completion, were summarized descriptively.

Results

Among 160 women, 54 (33.8%) deaths occurred during a median follow-up of 5.36 years. Treatment characteristics showed that 159 (99.4%) patients underwent surgery, 158 (98.8%) patients received chemotherapy, and 154 (96.3%) patients completed radiotherapy. Overall five-year OS was 66.3% (95% confidence interval (CI) = 58.4-73.0). Five-year OS was significantly higher in Stage I-II disease than in Stage III disease: 86.4% versus 45.6%, respectively (p < 0.001). Human epidermal growth factor receptor 2 (HER2)-enriched tumors, defined as hormone receptor-negative/HER2-positive disease, had the lowest observed five-year OS at 47.8%, compared with 72.3% for hormone receptor-positive disease and 62.8% for triple-negative breast cancer, although the unadjusted log-rank comparison did not reach statistical significance (p = 0.092). In multivariable analysis adjusted for stage, molecular subtype, age per 10-year increase, and tumor grade, Stage III disease (hazard ratio (HR) = 5.22; 95% CI = 2.66-10.26; p < 0.001) and HER2-enriched subtype (HR = 2.14; 95% CI = 1.06-4.32; p = 0.033) were independently associated with higher mortality.

Conclusions

In this single-center curative-intent cohort, Stage III disease was the strongest predictor of poorer five-year OS. HER2-enriched disease was also associated with worse adjusted survival, although this finding should be interpreted cautiously because HER2-directed therapy exposure and completion were not reliably captured. These results support efforts to improve earlier detection, timely referral, and access to evidence-based systemic therapy, including HER2-targeted treatment where clinically indicated.

## Introduction

Breast cancer is not a single biological entity but a group of diseases with varying receptor profiles, clinical behavior, treatment response, and prognosis. Immunohistochemical assessment of estrogen receptor, progesterone receptor, and human epidermal growth factor receptor 2 (HER2) status allows tumors to be grouped into clinically useful molecular categories, including hormone receptor (HR)-positive disease, HER2-enriched disease, and triple-negative breast cancer (TNBC) [1]. These categories are important because they influence treatment selection and are associated with different patterns of recurrence and survival [2].

Over recent decades, outcomes for breast cancer have improved because of earlier detection, multidisciplinary care, advances in systemic therapy, endocrine therapy, HER2-directed treatment, radiotherapy, and supportive care [3]. In high-income settings, these improvements are reflected in favorable population-level survival outcomes. For example, the American Cancer Society reports a five-year relative survival of approximately 91% for female breast cancer overall, with survival exceeding 99% for localized disease [4]. However, these outcomes are not equally achieved across all healthcare systems.

In Pakistan, breast cancer remains a major public health challenge. The National Cancer Registry at the National Institute of Health, Islamabad, identified breast cancer as the most frequently reported malignancy among women in its 2023 report [5]. Several local factors may contribute to poorer outcomes, including delayed presentation, limited awareness, financial barriers, social stigma, inconsistent access to diagnostic services, and restricted availability of some targeted treatments [6,7]. As a result, many patients present with locally advanced disease, where curative treatment is still possible but long-term survival is substantially reduced.

Although treatment options for breast cancer have expanded, their real-world impact depends on timely diagnosis, affordability, and access to standard therapy. In resource-limited settings, the availability of effective treatment does not always translate into equal survival benefit, especially when patients present late or cannot complete recommended systemic therapy. Therefore, local survival data are important for understanding outcome gaps and guiding service improvement.

Although the prognostic relevance of tumour stage and molecular subtype is well established, published Pakistani studies evaluating long-term overall survival (OS) by both variables remain limited. This study was therefore designed to assess real-world five-year OS among women with Stage I-III invasive breast cancer treated with curative intent at a single tertiary cancer center in Pakistan. We aimed to evaluate survival patterns by disease stage and molecular subtype and to generate locally relevant outcome data that may support earlier diagnosis, timely referral, and improved treatment planning.

## Materials and methods

Study design and setting

This retrospective cohort study was conducted at Cancer Care Hospital & Research Centre, Lahore, Pakistan, a tertiary cancer center providing multidisciplinary care for patients with breast cancer. Medical records were reviewed for women diagnosed with invasive breast carcinoma between January 1 and December 31, 2020.

Eligibility criteria

Patients were eligible for inclusion if they were female, aged 18 years or older, had histologically confirmed invasive breast carcinoma, and had American Joint Committee on Cancer (AJCC) 8th edition clinical Stage I-III disease without distant metastasis at diagnosis. Only patients whose curative-intent management and follow-up were recorded through our institute were included. Patients with de novo metastatic disease were excluded. Patients with insufficient follow-up information to calculate OS were also not included in the final analytic cohort.

Variables and definitions

Disease stage was recorded according to baseline clinical stage at diagnosis before definitive treatment. For survival analysis, tumor stage was grouped as Stage I-II and Stage III. Molecular subtype was assigned using immunohistochemistry results for estrogen receptor, progesterone receptor, and HER2 status. HR-positive disease included tumors positive for estrogen receptor and/or progesterone receptor, irrespective of HER2 expression. Therefore, HR-positive/HER2-positive tumors were analyzed within the HR-positive group. HER2-enriched disease was defined as HER2-positive disease with negative estrogen and progesterone receptors. TNBC was defined as estrogen receptor-negative, progesterone receptor-negative, and HER2-negative disease.

Treatment-related variables were extracted from available clinical records and manually verified where documentation was incomplete. These included type of surgery, receipt and timing of chemotherapy, and radiotherapy completion. Chemotherapy was categorized as neoadjuvant chemotherapy, adjuvant chemotherapy, or no chemotherapy given. Radiotherapy was categorized as completed, not indicated, or not completed because of death during neoadjuvant chemotherapy. Patients were classified according to treatment intent at presentation; patients who began curative-intent therapy but died before completing planned local treatment were retained in the cohort. Exact chemotherapy regimen, endocrine therapy adherence, and HER2-directed therapy exposure or completion were not consistently available and were therefore not analyzed as separate treatment variables.

OS was measured from the date of biopsy-confirmed diagnosis until death from any cause. Patients who were alive at last documented contact were censored on the date of their most recent follow-up. For the primary five-year OS analysis, follow-up time beyond five years from diagnostic biopsy was administratively censored at five years. The data cut-off date for vital status and follow-up ascertainment was October 27, 2025.

Statistical analysis

Data analysis was performed using SPSS version 29.0 (IBM Corp., Armonk, NY, USA). Continuous variables were summarized as medians with interquartile ranges, and categorical variables were summarized as frequencies and percentages. Treatment characteristics were summarized descriptively and were not included in the multivariable Cox regression model because exact chemotherapy regimen, endocrine therapy adherence, and HER2-directed therapy exposure were incompletely captured. OS was estimated using Kaplan-Meier analysis, and survival differences between groups were assessed with the log-rank test. Median follow-up was estimated using the reverse Kaplan-Meier method. Pointwise 95% confidence intervals (CIs) for five-year Kaplan-Meier survival estimates were calculated using log-log transformation.

Cox proportional hazards regression was used to estimate hazard ratios (HRs) with corresponding 95% CIs. The multivariable model was specified a priori and included tumor stage, molecular subtype, age per 10-year increase, and high tumor grade. Stage I-II disease was used as the reference category for stage, HR-positive disease was used as the reference category for molecular subtype, and Grade I-II disease was used as the reference category for tumor grade. A two-sided p-value of less than 0.05 was considered statistically significant.

Ethical approval

The study was approved by the Institutional Review Board of Cancer Care Hospital & Research Centre, Lahore, Pakistan (approval number IRB/CCH&RC/0508/25). All patient-level data were anonymized before analysis. The requirement for informed consent was waived by the Institutional Review Board because the study was retrospective and used de-identified clinical data.

## Results

A total of 171 women diagnosed in 2020 were screened. In total, 11 patients with de novo metastatic disease (M1/Stage IV) were excluded, leaving 160 women with Stage I-III, M0 invasive breast cancer for the final analysis. No additional patients were excluded for insufficient follow-up, missing survival time, or missing covariate data. Among the final cohort, 81 (50.6%) patients presented with Stage I-II disease and 79 (49.4%) had Stage III disease. The median age at diagnosis was 50 years (IQR = 40-57). HR-positive tumors were the most common subtype (n = 94, 58.8%), followed by HER2-enriched (n = 23, 14.4%) and TNBC (n = 43, 26.9%). Table 1 summarizes the baseline demographic and clinicopathologic characteristics of the study cohort.

**Table 1:** Baseline clinicopathologic characteristics overall and by molecular subtype. Values are shown as n (%) unless otherwise stated. Percentages in the molecular subtype columns are calculated within each subtype column. Stage I and Stage II were collapsed for survival analysis because of the small number of Stage I patients. The exact stage distribution in the overall cohort was: Stage I, n = 4 (2.5%); Stage II, n = 77 (48.1%); and Stage III, n = 79 (49.4%). Therefore, the combined Stage I–II survival estimate mainly reflects Stage II disease. HR = hormone receptor; HER2 = human epidermal growth factor receptor 2; TNBC = triple-negative breast cancer; IQR = interquartile range

Variable	Overall cohort, N = 160	HR-positive, n = 94	HER2-enriched, n = 23	TNBC, n = 43
Age, median (IQR), years	50 (40–57)	—	—	—
Disease stage				
Stage I–II	81 (50.6%)	46 (48.9%)	12 (52.2%)	23 (53.5%)
Stage III	79 (49.4%)	48 (51.1%)	11 (47.8%)	20 (46.5%)
Histologic grade				
Grade I–II	76 (47.5%)	51 (54.3%)	7 (30.4%)	18 (41.9%)
Grade III	84 (52.5%)	43 (45.7%)	16 (69.6%)	25 (58.1%)
Molecular subtype				
HR-positive	94 (58.8%)	—	—	—
HER2-enriched	23 (14.4%)	—	—	—
TNBC	43 (26.9%)	—	—	—

Treatment characteristics

Among the 160 patients in the primary M0 Stage I-III cohort, 125 (78.1%) underwent mastectomy and 34 (21.3%) underwent breast-conserving surgery. One (0.6%) patient died during neoadjuvant chemotherapy before surgery could be performed. Neoadjuvant chemotherapy was documented in 91 (56.9%) patients, adjuvant chemotherapy in 67 (41.9%) patients, and no chemotherapy was given in two (1.3%) patients. Radiotherapy was completed in 154 (96.3%) patients, was not indicated in five (3.1%) patients, and was not completed in one patient who died during neoadjuvant chemotherapy. Available treatment records showed that most patients received multimodality curative-intent treatment, as summarized in Table 2.

**Table 2:** Treatment characteristics of the primary M0 Stage I-III cohort. Values are shown as n (%). Percentages are calculated using the primary M0 Stage I-III cohort as the denominator. Treatment status was reported according to available records and manual verification. Exact chemotherapy regimen, endocrine therapy adherence, and human epidermal growth factor receptor 2-directed therapy exposure or completion were not consistently available in the retrospective dataset.

Variable	Overall cohort, N = 160
Surgery	
Mastectomy	125 (78.1%)
Breast-conserving surgery	34 (21.3%)
Surgery not performed; died during neoadjuvant chemotherapy	1 (0.6%)
Chemotherapy	
Neoadjuvant chemotherapy documented	91 (56.9%)
Adjuvant chemotherapy documented	67 (41.9%)
No chemotherapy given	2 (1.3%)
Radiotherapy	
Radiotherapy completed	154 (96.3%)
Radiotherapy not indicated	5 (3.1%)
Radiotherapy not completed; died during neoadjuvant chemotherapy	1 (0.6%)

Overall survival and follow-up

At a median follow-up of 5.36 years (95% CI = 5.17-5.55), calculated using the reverse Kaplan-Meier method, there were 54 (33.8%) deaths, and 106 (66.3%) deaths were censored. Median follow-up and person-years were calculated using full available follow-up, whereas Kaplan-Meier five-year OS estimates and Cox regression were based on OS time administratively censored at five years. The total person-years of follow-up, calculated using full available follow-up, were 690.45. The overall five-year OS for the cohort was 66.3% (95% CI = 58.4-73.0), and the median OS for the overall cohort was not reached at the time of analysis.

Survival by stage

**Figure 1:**
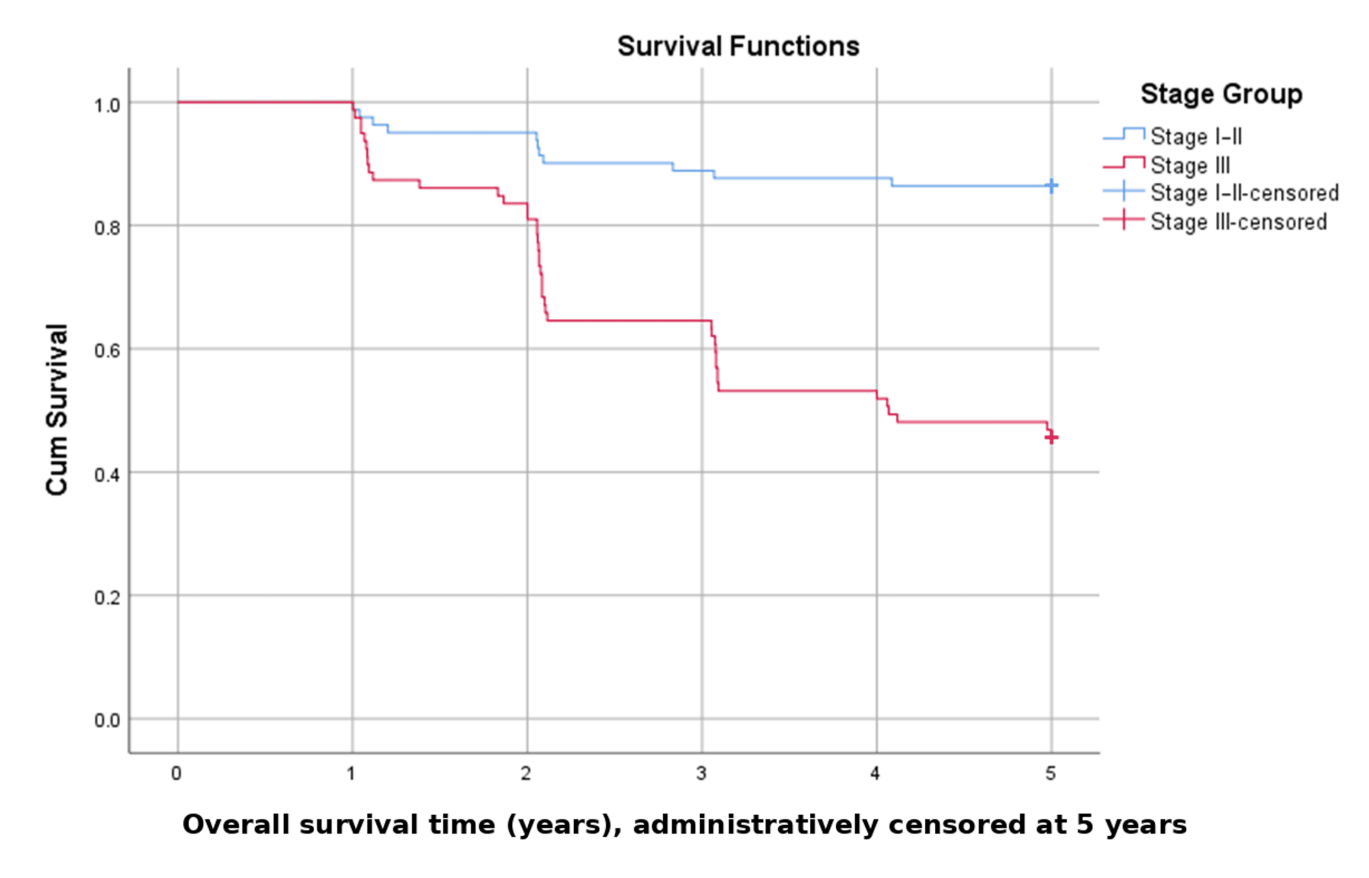
Kaplan–Meier curves showing overall survival by stage group. Stage I–II disease demonstrated significantly higher five-year overall survival compared with Stage III disease. Numbers at risk at zero, one, two, three, four, and five years were Stage I–II: 81, 81, 77, 72, 71, 69; Stage III: 79, 79, 66, 51, 42, 36. Generated using IBM SPSS Statistics for Windows, Version 29.0.

A significant difference in five-year OS was observed by disease stage. Five-year OS was significantly higher in Stage I-II disease than in Stage III disease: 86.4% (95% CI = 76.8-92.2) versus 45.6% (95% CI = 34.4-56.1), respectively (log-rank χ² = 29.02; p < 0.001). The Kaplan-Meier curves for OS by stage group are shown in Figure 1.

Survival by molecular subtype

**Figure 2:**
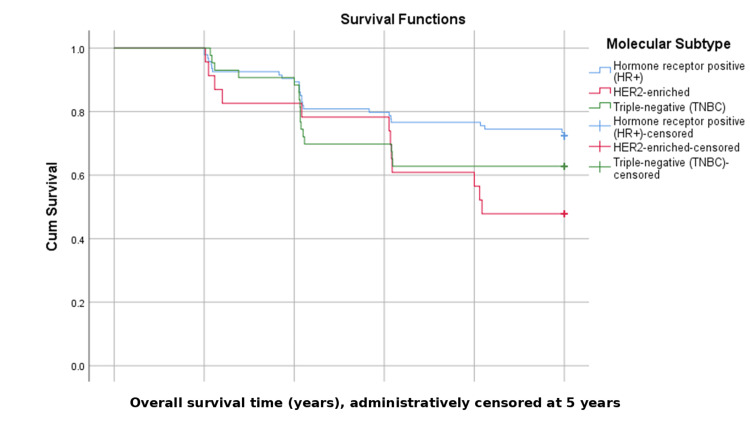
Kaplan–Meier curves showing overall survival by molecular subtype. HER2-enriched tumors showed the lowest observed five-year overall survival, whereas HR-positive disease showed the most favorable survival. Numbers at risk at zero, one, two, three, four, and five years were HR-positive: 94, 94, 85, 75, 72, 68; HER2-enriched: 23, 23, 19, 18, 14, 11; TNBC: 43, 43, 39, 30, 27, 26. HR = hormone receptor; HER2 = human epidermal growth factor receptor 2; TNBC = triple-negative breast cancer Generated using IBM SPSS Statistics for Windows, Version 29.0.

Five-year OS was 72.3% (95% CI = 62.1-80.2) in HR-positive disease, 47.8% (95% CI = 26.8-66.1) in HER2-enriched disease, and 62.8% (95% CI = 46.6-75.3) in TNBC. The difference did not reach statistical significance on log-rank testing (χ² = 4.78; p = 0.092), although HER2-enriched tumors showed the lowest observed five-year OS. The Kaplan-Meier curves for OS by molecular subtype are shown in Figure 2.

Five-year OS estimates with 95% CIs, event counts, censoring counts, and log-rank comparisons are summarized in Table 3.

**Table 3:** Five-year overall survival by stage group and molecular subtype. Pointwise 95% CIs for five-year Kaplan-Meier estimates were calculated using log-log transformation. OS = overall survival; CI = confidence interval; HR = hormone receptor; HER2 = human epidermal growth factor receptor 2; TNBC = triple-negative breast cancer

Comparison	Group	N	Deaths, n	Censored, n	5-year OS, %	95% CI	Log-rank χ²	P-value
Overall cohort	Overall	160	54	106	66.3	58.4–73.0	—	—
Stage group	Stage I–II	81	11	70	86.4	76.8–92.2	29.02	<0.001
Stage group	Stage III	79	43	36	45.6	34.4–56.1	29.02	<0.001
Molecular subtype	HR-positive	94	26	68	72.3	62.1–80.2	4.78	0.092
Molecular subtype	HER2-enriched	23	12	11	47.8	26.8–66.1	4.78	0.092
Molecular subtype	TNBC	43	16	27	62.8	46.6–75.3	4.78	0.092

Cox regression analysis

In multivariable Cox regression including tumor stage, molecular subtype, age per 10 years, and high tumor grade (Grade III vs I-II), Stage III disease was associated with substantially higher mortality compared with Stage I-II disease (HR = 5.22; 95% CI = 2.66-10.26; p < 0.001). Relative to HR-positive tumors, the HER2-enriched subtype had a higher hazard of death (HR = 2.14; 95% CI = 1.06-4.32; p = 0.033), whereas TNBC did not reach statistical significance (HR = 1.64; 95% CI = 0.86-3.14; p = 0.133). Age and tumor grade were not independently associated with OS (p = 0.785 and p = 0.571, respectively). The full multivariable Cox regression model is shown in Table 4.

**Table 4:** Multivariable Cox regression for predictors of overall survival. HR-positive disease was used as the reference category for molecular subtype. CI = confidence interval; HR = hormone receptor; HER2 = human epidermal growth factor receptor 2; TNBC = triple-negative breast cancer

Variable	Hazard ratio	95% CI	P-value
Stage III (vs Stage I–II)	5.22	2.66–10.26	<0.001
HER2-enriched (vs HR-positive)	2.14	1.06–4.32	0.033
TNBC (vs HR-positive)	1.64	0.86–3.14	0.133
Age (per 10-year increase)	1.03	0.82–1.30	0.785
Grade III (vs I–II)	1.18	0.67–2.07	0.571

## Discussion

The main contribution of this study is not the identification of tumor stage or molecular subtype as new biological prognostic factors, as these associations are already well established. Rather, this study provides real-world five-year OS data from a Pakistani tertiary cancer center, a low- and middle-income country (LMIC) setting where long-term breast cancer outcome data stratified by both stage and molecular subtype remain limited. In this curative-intent cohort, disease stage showed the strongest association with survival, with markedly poorer outcomes among patients presenting with Stage III disease. Although comparisons with international registry data should be made cautiously because Surveillance, Epidemiology, and End Results (SEER) localized and regional categories do not directly correspond to AJCC stage groups, the survival difference observed in our cohort provides local survival benchmarking and highlights the clinical impact of advanced-stage presentation in this treatment setting [8].

Almost half of the patients in this study had Stage III disease at presentation. This finding is important because it reflects a pattern frequently encountered in resource-limited oncology settings, where delayed diagnosis, low screening uptake, financial constraints, social stigma, and referral delays may all contribute to advanced-stage presentation. Similar barriers have been described in Pakistani breast cancer populations [6,7]. In our analysis, the survival gap between Stage I-II and Stage III disease was larger than the observed difference between molecular subtype groups, suggesting that earlier diagnosis may have a major effect on improving outcomes in this setting.

The treatment summary supports that the cohort largely received multimodality curative-intent care, with most patients undergoing surgery, chemotherapy, and radiotherapy where indicated. However, the comparatively low five-year survival should be interpreted in the context of the high proportion of Stage III disease, possible delays in diagnosis or referral, treatment-access constraints, and incomplete capture of systemic therapy details. In particular, HER2-directed therapy exposure and completion were not reliably captured, limiting causal interpretation of the poorer adjusted survival observed in the HER2-enriched subgroup.

Molecular subtype was also clinically relevant, although its association with survival was less prominent than that of stage. In unadjusted Kaplan-Meier analysis, survival differences across molecular subtype groups did not reach statistical significance. However, after adjustment for stage, age, and tumor grade, HER2-enriched disease was associated with a higher hazard of death compared with HR-positive disease. This difference between the log-rank and Cox regression findings is explainable because the log-rank test provides an unadjusted global comparison across all subtype groups, whereas the Cox model estimates adjusted hazards using a defined reference category. Nevertheless, the HER2-enriched subgroup was small, and this result should be interpreted with appropriate caution.

HER2-enriched tumors had the lowest observed five-year OS in this cohort. Access to HER2 testing and HER2-directed treatment remains an important issue in many resource-limited health systems [9]. Large clinical trials have shown that HER2-targeted therapy improves outcomes in both early and metastatic HER2-positive breast cancer [10,11]. In the present study, HER2-enriched disease was associated with poorer survival, but HER2-targeted therapy exposure and completion were not analyzed as separate treatment variables. Therefore, this finding should be interpreted as an association and not as definitive evidence that lack of HER2-targeted therapy alone caused the poorer outcome.

The observed survival pattern in the TNBC group should also be interpreted carefully. Although TNBC is generally considered an aggressive subtype, its outcome in a small retrospective cohort may be influenced by stage distribution, treatment selection, follow-up duration, and limited subgroup size [1,2]. In our cohort, Stage III disease was common across subtype groups, and Grade III disease was more frequent among HER2-enriched and triple-negative tumors. Tumor grade was not independently associated with OS in the multivariable model, but this should not be taken to mean that grade has no prognostic value. Rather, the non-significant result may reflect overlap between tumor grade, stage, molecular subtype, and the limited number of events.

These findings are consistent with broader global disparities in breast cancer outcomes. The World Health Organization Global Breast Cancer Initiative highlights that breast cancer mortality remains disproportionately high in LMICs, where late diagnosis and barriers to timely treatment are major contributors [12]. Pakistani registry data have also shown a substantial burden of advanced-stage breast cancer at diagnosis [13]. Practical strategies to improve outcomes should therefore include public awareness, accessible diagnostic pathways, timely referral, and improved access to evidence-based systemic therapy. Screening approaches, including clinical breast examination and mammography for appropriate age groups, may support earlier detection when implemented within accessible and sustainable health systems [14,15].

The strengths of this study include its focus on a curative-intent cohort, real-world follow-up, and use of both Kaplan-Meier survival analysis and multivariable Cox regression. The study also provides locally relevant survival data from Pakistan, where long-term outcomes stratified by both stage and molecular subtype remain underreported. However, several limitations should be acknowledged. This was a single-center retrospective study, and the findings may not be generalizable to all breast cancer populations in Pakistan. Although broad treatment categories were summarized, including surgery type, chemotherapy timing, and radiotherapy completion, the retrospective dataset did not consistently capture exact chemotherapy regimen, endocrine therapy adherence, HER2-directed therapy exposure or completion, treatment delays, affordability, socioeconomic status, or reasons for treatment omission. Therefore, treatment characteristics should be interpreted as contextual descriptors rather than variables proving guideline-concordant treatment or explaining survival causally.

The HER2-enriched subgroup was small, which reduced precision and produced wide CIs. In addition, HR-positive/HER2-positive tumors were included in the HR-positive group, meaning that the HER2-enriched category represented HR-negative/HER2-positive disease rather than all HER2-positive tumors. HER2 testing details, including confirmatory in situ hybridization or fluorescence in situ hybridization testing for equivocal immunohistochemical 2+ cases, were not uniformly available, which may have introduced subtype misclassification. Larger multicenter studies with complete systemic therapy data and standardized biomarker testing are needed to validate these findings and better define survival determinants in Pakistani breast cancer populations.

## Conclusions

This single-center Pakistani cohort provides real-world five-year OS data for women with M0 Stage I-III breast cancer treated with curative intent. Survival was substantially lower among patients with Stage III disease, highlighting the impact of advanced-stage presentation on outcomes. While the prognostic relevance of stage and molecular subtype is already established, this study contributes local survival benchmarking from an underrepresented LMIC setting. Available records showed that most patients received multimodality curative-intent treatment, but incomplete capture of exact systemic therapy, endocrine therapy adherence, and HER2-directed treatment limits causal interpretation. Larger multicenter studies with complete treatment exposure and socioeconomic data are needed to better define survival determinants in Pakistani breast cancer populations.
